# Supplementary material for: Mechanisms governing the pioneering and redistribution capabilities of the non-classical pioneer PU.1
Source: Nat Commun. 2020 Jan 21;11:402. doi: 10.1038/s41467-019-13960-2 (PMC6972792; doi:10.1038/s41467-019-13960-2)
Supplement: Supplementary file 7 — Source data [file 41467_2019_13960_MOESM7_ESM.zip › Source_Data/Figure6/Figure6B_MotifScanOutput/FLI1_PU.1/homerResults/motif19.similar.html]

motif19

## Information for motif19

T
A
G
C
G
C
T
A
A
C
T
G
C
G
T
A
A
C
G
T
C
G
T
A
T
C
G
A
T
A
C
G
T
C
G
A
  
Reverse Opposite:  

A
G
C
T
A
T
G
C
A
G
C
T
C
G
A
T
C
G
T
A
G
C
A
T
A
G
T
C
C
G
A
T
A
T
C
G
  

|  |  |
| --- | --- |
| p-value: | 1e-96 |
| log p-value: | -2.231e+02 |
| Information Content per bp: | 1.621 |
| Number of Target Sequences with motif | 1507.0 |
| Percentage of Target Sequences with motif | 15.15% |
| Number of Background Sequences with motif | 2867.6 |
| Percentage of Background Sequences with motif | 7.86% |
| Average Position of motif in Targets | 100.0 +/- 53.9bp |
| Average Position of motif in Background | 101.0 +/- 65.9bp |
| Strand Bias (log2 ratio + to - strand density) | 0.2 |
| Multiplicity (# of sites on avg that occur together) | 1.07 |
| Motif File: | file (matrix) reverse opposite |

### Similar de novo motifs found

|  |  |  |  |  |  |  |  |
| --- | --- | --- | --- | --- | --- | --- | --- |
| Rank | Match Score | Redundant Motif | P-value | log P-value | % of Targets | % of Background | Motif file |
| 1 | 0.879 | A G C T A T G C A C G T C G A T C G T A A C G T A G T C C G A T | 1e-92 | -212.310970 | 14.74% | 7.70% | motif file (matrix) |
| 2 | 0.893 | A G T C A C G T A C G T C G T A A C G T A G T C C G A T | 1e-89 | -206.714877 | 17.77% | 10.11% | motif file (matrix) |
| 3 | 0.877 | A C G T A T G C G A C T C G A T C G T A A C G T A G T C C G A T A G T C G C A T | 1e-62 | -143.977010 | 9.59% | 4.87% | motif file (matrix) |
| 4 | 0.838 | C T A G A G T C C G A T A T C G C G T A A G C T G C T A T G C A | 1e-42 | -98.739437 | 6.94% | 3.58% | motif file (matrix) |
| 5 | 0.793 | G C A T C A T G C G T A A G C T C T G A T G C A A T G C T C G A A C G T G A T C G C A T | 1e-26 | -62.057126 | 1.18% | 0.25% | motif file (matrix) |
